# *In vitro* and *in vivo* anticandidal activities of alginate-enclosed chitosan–calcium phosphate-loaded Fe-bovine lactoferrin nanocapsules

**DOI:** 10.4155/fsoa-2017-0085

**Published:** 2017-11-16

**Authors:** Khoo Miew Leng, Soundararajan Vijayarathna, Subramanion L Jothy, Sreenivasan Sasidharan, Jagat R Kanwar

**Affiliations:** 1 Institute for Research in Molecular Medicine (INFORMM), Universiti Sains Malaysia, USM 11800, Pulau Pinang, Malaysia; 2 Nanomedicine-Laboratory of Immunology & Molecular Biomedical Research (LIMBR), School of Medicine (SoM), Faculty of Health, Deakin University, Waurn Ponds, Geelong, VIC 3217, Australia

**Keywords:** antiyeast activity, *Candida albicans*, electron microscopy, histopathology, lactoferrin, nanocapsules

## Abstract

**Aim:**

To study the *in vitro* and *in vivo* anticandidal activity of nanocapsulated bovine lactoferrin.

**Materials & methods:**

*In vitro* and *in vivo* antimicrobial activities were conducted to study the anticandidal activities of nanocapsules (NCs).

**Results:**

The NCs showed good anticandidal activities. The disruption of cell wall and cell membrane was noted via microscopy studies. The NCs changed the normal growth profile of *Candida albicans*. NCs reduced the colony forming unit in kidney and blood samples. Histopathological examination showed better cell structure and coordination compared with untreated mice kidney. NCs also enhanced the natural killing properties of *C. albicans* by epithelial cells.

**Conclusion:**

NCs have effective anticandidal properties and have the potential as a therapeutic agent against candidiasis.

## Lay abstract

Previous study revealed that lactoferrin had potent anticandidal action against *C. albicans*. However, encapsulated lactoferrin has never been tested for anticandidal activity in detail. In the present study, we evaluate nanocapsulated lactoferrin for anticandidal effects. To observe the anticandidal properties of encapsulated lactoferrin, various studies were conducted. Our findings showed that encapsulated lactoferrin demonstrates remarkable efficacy against *C. albicans*.

## Graphical abstract

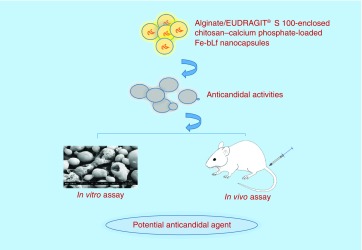

Pathogenic fungi such as *Candida albicans* are a key contributory agent for opportunistic infections. Immunocompromised individuals such as AIDS patients are at danger for opportunistic infections, which if not cured properly, contribute to the death linked to their disorders. Inadequate availability of effective antifungal drugs and the increasing prevalence of this life threatening fungal infection [1] have conspired to outgrow a necessity for developing novel treatment options for opportunistic infections by pathogenic fungi such as *C. albicans*. Fungal cells which categorized as eukaryotes shared numerous similarities with mammalian cells such as the nucleus contains DNA organized into chromosomes with distinct cytoplasmic organelles and biosynthetic pathways similar to mammalian cells. These likenesses have made extra problems in the design of antifungal agent with selective toxicity to fungal cells [2,3]. Moreover, fungal cells, like various microorganisms, are surrounded by cell wall that consists of β-glucan, chitin and mannoproteins, which offer physical support and defense to the cell and develop additional obstacle in the design of antifungal agent [4]. The presence of complex biopolymer fungal cell wall, which provides the physical protection of the yeast cell make the discovery of antifungal agents remains an important scientific challenge. The insoluble polysaccharides in cell wall convey mechanical strength to the fungal cell [5]. Hence, the fungal cell wall is emerging as an important better defined therapeutic target. For example, in *C. albicans*, it has been proposed that β-glucan and chitin are related to the strength and shape of the cell wall, while mannoproteins are accountable for the porosity, its antigenicity and adhesion [5]. As a result, no antifungal drug was available for the cure of fungal infections until the finding of amphotericin B in 1953 [6]. This was followed by the development of many new antifungal agents. However, with the advent of the immunocompromised diseases such as AIDS, the growing number of patients alive with repressed immune systems and the deficiency of effective antifungal agent for the cure of fungal infections, there has been renaissance in the awareness in the search for new, safer, and more efficacious antifungal agents to battle serious fungal infections [7,8].

Concomitant with this, most of the consideration has been dedicated to the development of an ideal antifungal agent. In so doing, an ideal antifungal agent should meet some criteria such as directed at a specific fungal target, have multiple delivery methods, mainly oral availability with minimal toxicities or side effects. It is clear that designing an antifungal drug that fulfills the above criteria is a key challenge and requires the identification of new biological natural products such as lactoferrin. Lactoferrin is a multifunctional iron-binding glycoprotein belonging to the transferrin family present in mammalian milk. It has also been shown to inhibit a wide range of fungal, bacterial, viral and parasitic pathogens [9–12]. The anticandidal mode of action of lactoferrin was suggested to be due to cell wall perturbation [9], as proved by cryo-scanning electron microscopy study which showed the drastic changes in the cell wall, leading to the formation of surface blebs, swelling and cell collapse [13]. Similar damages on cell wall were also reported by Nikawa *et al.* [14,15] after *Candida* exposure to both human and bovine lactoferrin and it was concluded that the anticandidal activity of lactoferrin is due to direct contact of the protein with the fungal cell surface [16]. However, encapsulated lactoferrin has never been tested for anticandidal activity in detail. The successful administration of glycoprotein such as lactoferrin by oral route with maintaining their active conformation remains a key task in the field of pharmaceutical technology. In the present study, we propose the use of nanoencapsulated-based system for effective oral route-based delivery system of acid-labile lactoferrin. Thus in the present study, the anticandidal activity of encapsulated lactoferrin was tested in *in vivo* in well-established mice model and *in vitro* models.

For the past few decades, various formulations of nanoparticles have been used for drug delivery research to increase therapeutic benefit [17]. For the current study, the *in vitro* and *in vivo* experiments were carried out by using an encapsulated Fe-bovine lactoferrin (Fe-bLf; AEC-CP-Fe-bLf nanocapsules [NCs]) preparations in the treatment of *C. albicans*. Alginate-enclosed chitosan–calcium phosphate-loaded Fe-bLf NCs were prepared by a combination of nanoprecipitation and ionic gelation methods by Kanwar *et al.* [18] with modifications. Interestingly, in this study for the first time, the efficacy of encapsulated Fe-bLf (NCs) as an antifungal agent was assessed, especially in the case of *C. albicans.* Candidiasis is a fungal infection caused by 20 types of *Candida* species and most commonly by the dimorphic fungus *C. albicans*. In contrast to other fungal pathogens, *C. albicans* is a member of the normal flora in the digestive system, respiratory system, vaginal area and mouth. *C. albicans* do not produce any disease in healthy individuals. The growth of *C. albicans* is suppressed by other normal flora in the body. However, if the normal flora is being disturbed, *Candida* can multiply rapidly and cause candidiasis. In recent years, *Candida* species have also been identified as nosocomial pathogens [19]. Besides that, *Candida* can also be transmitted sexually. A majority of candidal infections involves the skin or mucous membranes. This is because *C. albicans* is a strict aerobe and therefore such surfaces are suitable for its growth. Previously, lactoferrin was reported to possess antifungal properties by Kirkpatrick *et al.* [20]. Oral candidiasis is linked with impaired secretion of lactoferrin by the salivary glands. For instance, in one study patients had a reduction as much as 65% in lactoferrin as compared with healthy individuals. Moreover, nanoformulation of lactoferrin offers various advantages such as sustained release, better efficiency, lesser side effects, target specificity and improved delivery [21]. Therefore, the current study was conducted to evaluate the anticandidal activity of encapsulated lactoferrin (NCs).

## Materials & methods

### Preparation of endotoxin-free Fe-bLf

Endotoxin-free bLf was prepared from Australian bovine milk [22]. Endotoxin was evaluated by using Genscript ToxinSensor™, Chromogenic Limulus Amebocyte Lysate Endotoxin Assay Kit (Genscript ToxinSensor, NJ, USA). Ferum-bLf was produced from bLf based on the previously described method established in Deakin’s laboratory (VIC, Australia). Following treatment with mild acidic solution (pH 2.6), bLf was dialyzed for a duration of 48 h in 0.1 M citric acid to remove bound metal ions and then saturated with ferric [Fe(III)] nonahydrate to form bLf–Fe^3+^co-ordinate complexes to produce deep-red colored Fe-bLf. The Fe-bLf produced was loaded on to the chitosan–calcium phosphate NCs and coated with alginate.

### Preparation of alginate/EUDRAGIT^®^ S 100-enclosed chitosan–calcium phosphate-loaded Fe-bLf NCs

Alginate/EUDRAGIT^®^ S (Rohm Pharmaceuticals, Darmstadt, Germany) 100-enclosed chitosan–calcium phosphate-loaded Fe-bLf NCs were produced by a combination of nanoprecipitation and ionic gelation methods [18,23]. Calcium phosphate was prepared from its constituents by adding disodium hydrogen orthophosphate (Na_2_HPO_4_) in a molar ratio of 4:1 to calcium chloride (CaCl_2_) in a dropwise manner while stirring continuously. The suspension was sonicated at 4°C to ensure a white precipitate of calcium phosphate was obtained. The calcium phosphate (1% w/v) suspension produced was incubated for 24 h with 10% w/w Fe-bLf (from Section 3.2.1) with constant stirring at 4°C, at a pH ≤ 8.0, the isoelectric pH of lactoferrin, to adsorb Fe-bLf onto the NCs. Following the electrostatic interaction of Fe-bLf on calcium phosphate (ceramic core), the suspension formed was centrifuged and washed several times to eliminate traces of unbound protein and then freeze-dried. 0.01% w/w chitosan solution in acetate buffer (pH 4) was added to calcium phosphate under constant stirring [18]. Then, 0.01% of cross linking agent, sodium tri-polyphosphate was added drop wise. Constant stirring at 6000 r.p.m. (MSH-20D, Laboratory Instruments) for 12 h was performed to ensure that the nanoformulation attained the size of 200 ± 40 nm, followed by freeze drying to ensure spherical shape of the samples is acquired. These nanocores were then coated with alginate gel by using 2% w/v EUDRAGIT S 100/alginate solution and calcium chloride, with 0.6% mass ratio of calcium alginate. Finally, the nanocarriers formed were washed and lyophilized. All these experiments were conducted at 4°C as to protect the polymeric and protein constituents in the formulation.

### *Candida albicans* USM-K1

Yeast isolate used in this study was *C. albicans* USM-K1 obtained from the Microbiology Department of Universiti Sains Malaysia Hospital, Kelantan (HUSM). These yeast strain was isolated from patient. The yeast strain was stored in 50% glycerol stock at -80°C to maintain their long-term viability. For all the experiments, the yeast strains were subcultured for single colonies on Sabouraud dextrose agar (SDA) and incubated at 37°C for 18 h in an incubator (Loading Modell 100–800, Memmert, Schwabach, Germany).

### *C. albicans* inoculum preparation

Inoculum size is very important and has to be standardized at a certain value to obtain reliable, reproducible and significant results. Therefore, inoculum size was standardized throughout this study. A loop (25 μl) of yeast was obtained from a pure single colony from SDA and was suspended in 10 ml of Sabouraud dextrose broth (SDB; HiMedia, Mumbai, India). The broth was incubated overnight at 37°C at 200 r.p.m. using an orbital shaker (Model 420, Forma Orbital Shaker, Thermo Fisher Scientific, MA, USA). Then, the culture was standardized to OD_600_ = 0.3 using spectrophotometer (Thermo Fisher Scientific) [24].

### Disk diffusion method

Antimicrobial activity was determined by modification of the disk diffusion method by [24]. Paper disk (Advantec 90 mm, Toyo Roshi Kaisha, Ltd, Tokoyo, Japan) with a diameter of 6 mm was sterilized by autoclaving at 121°C for 15 min and kept at room temperature until used. A 100 μl of mid exponential phase yeast cultures (OD_600_ = 0.3) was spread onto SDA and left to dry for 1 h at room temperature. Then, the sterile disk was placed on the surface of the plates. Sterile paper disks was impregnated with 20 μl NCs (2 mg/ml). Deionized water was used as a negative control. Miconazole nitrate (30 μg/ml; Duchefa Biochemie, Haarlem, The Netherlands) was used as a positive control. The plates were incubated in an incubator (Memmert) for 18 h at 37°C. The test was conducted in triplicate. Antimicrobial activity was determined by measuring the diameter of inhibition zone around the disk.

### Minimal inhibitory concentration

MIC was determined based on the modification of the method of Håversen *et al.* [25]. Microdilution method was conducted using 96-well microtiter plate. A 96-well plate was filled with 130 μl of SDB in all the wells. 150 μl of deionized water was added in the first well of the first row and a twofold serial dilution was performed to serve as a negative control. Subsequently, 150 μl of 10 μg/ml miconazole nitrate was added in the first well of second row and a twofold serial dilution was conducted to serve as positive control. After that, 150 μl NCs were added in the following row and a twofold serial dilution was performed. Then, 20 μl mid exponential phase yeast cultures (OD_600_ = 0.3) were added in all the wells. The plates were incubated at 37°C for 24 h in the incubator (Memmert). The experiment was conducted in triplicate. The MIC was considered as the lowest concentration of NCs whereby observable growth was inhibited. The results were confirmed by reading the absorbance of the plate at the wavelength of 600 nm using an ELISA microplate reader (Multiskan Spectrum, Thermo Scientific, Vantaa, Finland).

### Time–kill study

Time–kill study was conducted according to the method of Sasidharan *et al.* [26] with slight modifications. In order to evaluate the anticandidal effect of NCs with various MIC concentrations over time, growth profile curves were plotted. NCs were prepared at a volume of 24 ml in 100 ml Erlenmeyer flask at concentrations of 0.5 MIC, MIC, 2 MIC and 4 MIC using SDB. 1 ml inoculum of yeast culture that has been standardized to OD_600_ = 0.3 was inoculated into the flasks with various concentrations of NCs. The final concentrations of NCs were maintained. 1 ml of inoculum was added into SDB without NCs as the control group. The experiment was conducted in triplicate. The flasks were incubated at 37°C at 200 r.p.m. using an orbital shaker (491 Forma Incubated/Refrigerated Stackable Orbital Shaker, Thermo Scientific) for 48 h. The growth profile was studied for 48 h. Sampling was done every 4 h whereby 1 ml of culture was pipetted out aseptically from the Erlenmeyer flasks starting from 0 h. Absorbance reading was taken using spectrophotometer (Biomate 3, Thermo Spectronic, NY, USA) at the wavelength of 600 nm (OD_600_). SDB without NCs was used as the blank. After that, a graph was plotted to determine the effect of NCs on the growth profile of *C. albicans*.

### Morphological changes of *C. albicans* after treatment with NCs

A volume of 100 μl mid exponential phase yeast cultures at OD_600_ = 0.3 was spread onto SDA and incubated for 6 h at 37°C. After that, a volume of 20 μl NCs (2 mg/ml) was dropped onto the agar surface. The plates were swirled, spread with glass rod and incubated at 37°C for 12, 24 and 36 h [27]. Normal cells that were vehicle treated with deionized water only were used as the control. After the incubation of the plates at the stated duration, the following tests were performed.

### Observation of *C. albicans* morphological changes by scanning electron microscope

Scanning electron microscope (SEM) sample preparation was based on the Electron Microscopy Protocols of Electron Microscopy Unit of School of Biological Sciences, Universiti Sains Malaysia. A planchette was prepared with double-sided sticky tape. The sample positions were labelled carefully. A small piece of agar containing yeast growth (about 5 mm × 5 mm) was cut and quickly placed on the double sided sticky tape. Cubes of agar were taken out from the vehicle treated (control), 12, 24 and 36 h plates. The planchette was then placed in a filter paper lined petri dish. The filter paper was wet with a few drops of 2% osmium tetroxide and the petri dish was closed immediately. The petri dish was left in the fume hood for about 1–2 h. This process is known as ‘vapor fixation'. Once the sample is fixed, the planchette is plunged into slushy nitrogen (-210°C) and transferred to the ‘peltier-cooled’ stage of the freeze dryer (Emitech K750, Emitech, Ashford, UK) and left to freeze dry for about 10 h. After that, the planchette containing the samples has to be kept in a desiccator if not viewed immediately. The samples were sputtered with approximately 5–10 nm of gold before observation using scanning electron microscope (Leo Supra 50 VP Field Emission SEM [FESEM], Carl-Ziess SMT, Oberkochen, Germany).

### Observation of *C. albicans* morphological changes by transmission electron microscope

McDowell–Trump fixative was dropped onto the agar surface of the vehicle treated (control), 12, 24 and 36 h plates to fix the yeast cells. The yeast cultures were scraped from the plates and placed into the microcentrifuge tubes. A small amount of fixative was added into the microcentrifuge tubes. The eppendorf tubes were centrifuged (Sigma 1-141, Eppendorf, Sartorius, Germany) at 1000 ×*g* for 5 min. The supernatant was discarded and the pellet was resuspended with the fixative. Subsequently, the sample was wrapped using a parafilm to avoid dehydration and kept in the cold room at 4°C until further use. Processing of the yeast samples for observation by a transmission electron microscope (TEM) was conducted according to the Electron Microscopy Protocols of Electron Microscopy Unit of School of Biological Sciences, Universiti Sains Malaysia. The sample was centrifuged at 1000–2000 ×*g* for 15 min. The supernatant was discarded and the pellet resuspended with McDowell–Trump fixative prepared in 0.1 M phosphate buffer (pH 7.2) for at least 2 h for fixation of the cells. The resuspended sample was then centrifuged and the supernatant discarded. The pellet was resuspended in 0.1 M phosphate buffer (buffer wash 1). The resuspended sample was centrifuged. The supernatant discarded and resuspended in 0.1 M phosphate buffer (buffer wash 2). The resuspended sample centrifuged. The supernatant discarded and the pellet resuspended in 1% osmium tetroxide prepared in phosphate buffer for 1 h (post-fixation). The resuspended sample was centrifuged. The supernatant discarded and the pellet resuspended in distilled water (post-fix wash 1). The resuspended sample was centrifuged. The supernatant discarded and the pellet resuspended in distilled water (post-fix wash 2). The resuspended sample was centrifuged, the supernatant discarded and the tubes containing the pellet of fixed cells were placed in a waterbath at 45°C for about 15–20 min depending on the quantity of sample.

A 3% solution of agar was prepared by dissolving the agar in boiling distilled water. The solution was poured into a test tube while it is still molten and placed in the water bath at 45°C. The agar remains liquid at this temperature. After the temperature of both the agar and the pellet has equilibrated to 45°C, a small drop of agar was transferred to the tube containing pellet of cells using a warm pipette. The pellet was stirred just sufficient to break the pellet into small blocks and to be suspended in the agar. The agar with the suspended pellet was poured immediately to a glass microscope slide. After that the agarose was placed in a refrigerator for approximately 1–2 min until the agarose solidifies. Subsequently, the solidified agar contains the cells were cut into small cubes of about 1 mm^3^ with a sharp razor blade and then placed in a vial containing 50% ethanol. These cubes were then processed in the same manner as pieces of a cohesive pellet or tissue. Dehydration was carried out at the following duration: 50% ethanol (15 min), 75% ethanol (15 min), 95% ethanol (15 min) twice, 100% ethanol (30 min) twice and 100% acetone (10 min) twice. The resin–acetone (1:1) mix was infiltrated into the samples in a rotator followed by infiltration in Spurr's mix overnight in the rotator. Subsequently, the samples were infiltrated in a new change of Spurr's mix for another 5 h in the rotator. Then, embedding was performed by insertion of samples in melted resin before the samples were cured at 60°C for 12–48 h. After processing, the samples were then sectioned using an ultramicrotome. Ultra-thin sections were stained with uranyl acetate and lead citrate and observed under TEM (Energy-filtered TEM Libra 120, Carl Zeiss).

### Germ tube formation

Germ tube formation was conducted according to the modification of the method of Ghalehnoo *et al.* [28]. *C. albicans* were cultured on a SDA plate overnight at 37°C in the incubator (Memmert). One colony was resuspended in 1 ml of fetal bovine serum and incubated in the waterbath at 37°C for 1 to 2 h. Control contained fetal bovine serum only without NCs. One drop was taken out using micropipette, placed on a microscopic slide and observed under the phase contrast microscopy at 400× magnification (compound light microscope, Olympus BX41; Olympus Optical Co. Ltd, Tokyo, Japan).

### *In vivo* anticandidal activity

#### Laboratory animals

Swiss albino mice (male) weighing between 25 and 35 g were obtained from the Animal House, University Science Malaysia (USM), Penang. The cages containing mice were placed in the Animal Room of Institute for Research of Molecular Medicine (INFORMM), Universiti Sains Malaysia which is 24 h air-conditioned. Pellets and water were given to mice *ad libitum*. The experimental protocols of this study were approved by the Animal Ethics Committee USM (AECUSM) [USM/Animal Ethics Approval/2016/(723)]. Procedures of animal handling were carried out in accordance to the internationally accepted principles for laboratory animal use and care. Animals were kept in the cages for at least 1 week to allow acclimatization to the animal room conditions.

### *In vivo* anticandidal assessment

A volume (0.1 ml) of 1 × 10^7^ *C. albicans* cells/ml in phosphate-buffered saline (PBS) was injected intravenously in the lateral tail vein of mice [29]. The mice were separated into two groups of six each and given treatment as described in Table 1. All the mice were sacrificed by cervical dislocation on the fifth day after *C. albicans* was inoculated intravenously.

**Table 1.** **Experimental groups and types of treatments for *in vivo* anticandidal activity study of nanocapsules.**

**Experimental group**	**Type of treatments**
Group 1 (control)	iv. *C. albicans*: 24 h gap, followed by treatment with PBS (intraperitoneal [ip.] once-daily for 3 days)

Group 2 (curative)	iv. *C. albicans*: 24 h gap, followed by treatment with NCs, 2.5 g/kg body weight (ip. once-daily for 3 days)

NC: Nanocapsule; PBS: Phosphate-buffered saline.

A technique for plating organ homogenates which allows the quantitative determination of fungal populations from the kidney of infected mice was conducted [30]. A volume of 0.1 ml of blood was withdrawn from the renal artery with 0.1 ml of heparin (25 U/ml) as an anticoagulant added into the blood sample. Then the kidneys of each mice were removed aseptically. The kidneys (1 g) were then placed into sterile centrifuge tubes and homogenized using pestle and mortar in 5 ml of sterile PBS. Serial dilution of the homogenate and blood samples were conducted. A total of 0.1 ml from each dilution was pipetted on the surface of three SDA agar plates and spread evenly. The plates were incubated at 37°C for 24 h. The experiment was conducted in triplicate. The colonies were then enumerated and the colony forming unit (CFU) was calculated per gram of organ and per milliliter of blood sample, respectively. The numbers of colonies from the control and the curative group were compared using *t*-test by using SPSS 22.0 software for Windows. Values of p < 0.05 were considered significant.

### Histopathological examination

After the mice were sacrificed, the kidneys were collected for histological examination [31]. Pieces of tissue not more than 5 mm thick were cut from the kidneys. The tissues were then fixed in 10% buffered neutral formalin for up to 24 h. Then, the tissues were dehydrated in three changes of 96% alcohol, for 2 h each change (ten-times volume of tissue) and three changes of absolute alcohol, 2 h each change. The tissues were left overnight in xylene. Subsequently, the tissues were transferred to fresh xylene until the tissues become transparent and clearing was completed. Then, the tissues were placed in melted paraffin, three changes of 1 h each. After that, the tissues were embedded in paraffin. The paraffin blocks were trimmed and placed on the object holder of the microtome (Leica Camera AG, Wetzlar, Germany) for sectioning the blocks into 5–6 μm sections. The sections were then floated flat on the floatation bath. Single sections were floated onto glass slides. Excess water was removed and sections were dried in oven at 50–55°C for at least 1.5 h. Sections were removed from oven and immersed in two changes of xylene each of 5 min duration. Then, the sections were immersed in two changes (3 min each) of absolute alcohol, two changes (3 min each) of 95% alcohol and then placed in tap water. The tissue sections were stained in Harris’ hematoxylin for 3–5 min. The sections were differentiated in acid alcohol and blue 5–10 min in tap water. Sections were counterstained in 0.5% eosin and 70% alcohol for 2–3 min. After that, tissue sections were dehydrated in two changes of 95% alcohol rapidly and left in two changes of absolute alcohol (3 min each). Sections were cleared in two changes of xylene (3 min each) and mounted in Permount. A comparison of the microscopic features of the kidneys of mice from Group 1 and Group 2 was conducted.

### Oral epithelial cell anticandidal activity

#### Isolation of oral epithelial cells

10 ml of unstimulated saliva was expectorated from a human subject into a sterile polypropylene tube (Labserv, Warrington, UK) [32]. The sample was centrifuged (5804 R, Eppendorf) at 800 ×*g* for 5 min. After washing with sterile PBS, the cell pellet was resuspended in Hanks’ balanced salt solution (Corning, NY, USA) and passed over a 20 μm sterile nylon membrane. The epithelial cells retained on the nylon membrane were washed and resuspended in cryopreservative (50% fetal calf serum, 25% Roswell Park Memorial Institute (RPMI) 1640 medium and 15% dimethylsulfoxide) and stored at -70°C until use.

#### Target cells

*C. albicans* USM-K1 was grown on SDA at 37°C. One colony was used to inoculate 10 ml of SDB and incubated for 18 h at 37°C. The yeast cells were enumerated on a hemacytometer using trypan-blue exclusion. The method was modified from Nomanbhoy *et al.* [33].

#### Vital staining of *C. albicans* in the coculture

*C. albicans* untreated with NCs (control), coculture of oral epithelial cells and *C. albicans* (1:2.6 ratio) and coculture of oral epithelial cells and *C. albicans* (1:2.6 ratio) treated with NCs each in a volume of 2 ml/well were incubated in a 24-well plate (Costar 3526, Corning). After 1-h incubation at room temperature, the cocultures were collected and washed twice. Similarly, the untreated *C. albicans* were also collected and washed twice. Fluorescein diacetate (50 μg/ml which stains living cells; Tokyo Chemical Industry Co. Ltd, Tokyo, Japan) and propidium iodide (1 μg/ml which stains dead cells; Sigma–Aldrich, Saint-Quentin-Fallavier, France) were added simultaneously to the cell pellets and left in the dark for 30 min at room temperature. Following the incubation, the cocultures were washed with PBS by centrifugation at 800 ×*g* for 5 min, later by 20% fetal bovine serum and then washed again with PBS. Finally, the pellets were resuspended in 100 μl of PBS and 5 μl of each coculture was placed on a slide and observed under a dual-filter fluorescent microscope (Fluo BX53 Cell Sens, Olympus BX53, Olympus Optical Co. Ltd). Blue light was selected as the suitable wavelength for viewing. Controls for vital staining included the living and dead (heat-killed) *C. albicans* in the presence and absence of oral epithelial cells. The method was modified from Nomanbhoy *et al.* [33].

## Results

### Determination of *in vitro* antimicrobial activity of NCs

The antimicrobial potential of NCs was assessed against *C. albicans* in terms of the zone of inhibition of yeast growth. NCs show significant inhibition zone against *C. albicans*. The diameter of the inhibition zone measured for *C. albicans* is 18.33 mm. The zone of clearance produced by the commercial antibiotic miconazole disk is larger than that produced by the NCs disk (p < 0.05).

### Minimal inhibitory concentration of NCs

**Figure 1.**
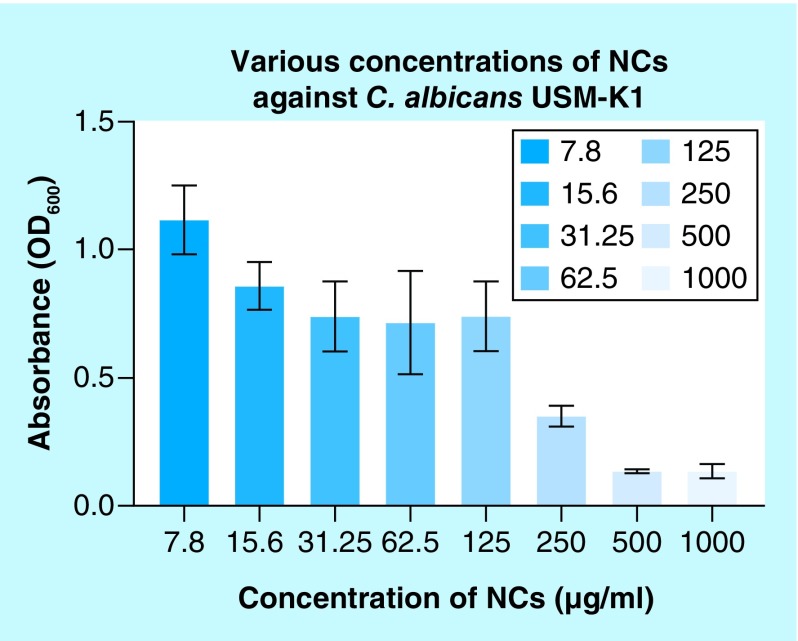
**Minimal inhibitory concentration (500 μg/ml) of nanocapsules against *Candida albicans*.** NC: Nanocapsule; OD: Optical density; USM: Universiti Sains Malaysia.

The lowest concentration of NCs solution that can inhibit the growth of *C. albicans* is determined as MIC. NCs show MIC value of 500 μg/ml against *C. albicans* in this study (Figure 1). The lowest concentration of peptide that completely inhibits the growth of bacteria by visual inspection or growth percentage that was less than 5% of negative control detection spectrophotometrically is defined as an MIC [34]. This is also applicable to yeast such as *C. albicans*.

### Fungicidal effect of NCs at various concentrations over time

**Figure 2.**
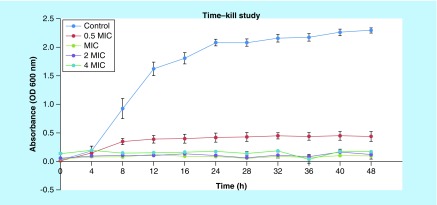
**Growth profile for *Candida albicans* in Sabouraud dextrose broth with 0 (control), 250 μg/ml (½ MIC), 500 μg/ml (MIC), 1000 μg/ml (2 MIC) and 2000 μg/ml (4 MIC) concentration of nanocapsules.** OD: Optical density.

Time–kill study was conducted to assess the fungicidal effect of NCs at 0.5 MIC (250 μg/ml), MIC (500 μg/ml), 2 MIC (1 mg/ml) and 4 MIC (2 mg/ml) over time. The result of the time–kill curves for *C. albicans* is shown in Figure 2. The control without the treatment of NCs, showed a normal growth curve with various growth phases such as a lag phase, a log phase and a stationary phase. However, at 1/2 × MIC, NCs demonstrated a large drop in optical density (OD) reading after 8 h, which led to the stationary phase of the yeast growth. At MIC, 2 × MIC and 4 × MIC, NCs produce absolute yeast eradication after only 4 h. The time–kill study showed the effectiveness of NCs as an anticandidal agent against *C. albicans* over a duration of 48 h.

### Anticandidal mode of actions of NCs

**Figure 3.**
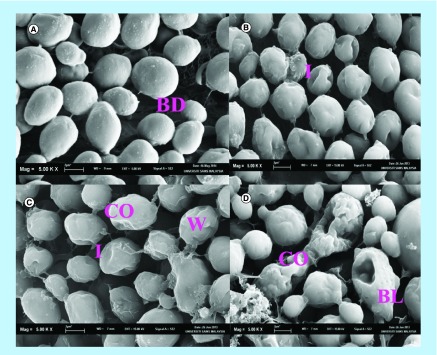
**Scanning electron microscopy micrograph of the untreated and nanocapsule-treated cells of *Candida albicans*.** **(A)** Control cells of *C. albicans*; **(B)** 12 h *C. albicans* cells treated with 2 mg/ml of NCs; **(C)** 24 h *C. albicans* cells treated with 2 mg/ml of NCs and; **(D)** 36 h *C. albicans* cells treated with 2 mg/ml of NCs. ×5000 magnification used for all images. BD: Budding; BL: Blebbing; CO: Collapse; I: Indentation; NC: Nanocapsule; W: Wrinkle.

Electron microscope studies were conducted to observe the effects of NCs on the extra and intramorphology of *C. albicans*. The effects of NCs (2 mg/ml) on the extramorphological features of *C. albicans* were observed using a SEM and a TEM. 4 × MIC (2 mg/ml) was chosen for electron microscopy studies to observe the anticandidal mechanism of NCs on *C. albicans* and also to obtain clearer images. The SEM study shows that the NCs have the ability to cause morphological and structural changes of *C. albicans* (Figure 3). Vehicle-treated yeast cells are treated with deionized water only (control). The vehicle-treated yeast cells (control) were not altered by the NCs treatment with normal cell morphology. The control cells were oval and demonstrated budding which was a characteristic of the yeast cells (Figure 3A). Alteration of *C. albicans* morphology started to take place after 12 h of treatment of NCs. The yeast cells started to get close to each other and were arranged in groups after 12 h of NC treatment. The cells’ morphology was altered whereby the surface of cells showed indentations and signs of destruction (Figure 3B). After 24 h of NC treatment, the extent of destruction of yeast cells was more significant. Indentations on the cell surface were more prominent as the cells were severely damaged. The cells appeared more wrinkled. This is due to cell wall perturbation and cell membrane disruption causing the collapse of cells (Figure 3C). After 36 h of treatment with NCs, the yeast cells lost their original surface ultrastructure and the cells appeared uneven. The yeast cells were completely destroyed. Very deep indentations were observed on the cell surface. Blebbing and cell collapse was also observed (Figure 3D). At this stage, the yeast cells had lost their metabolic functions resulting in cell death. This shows that NCs caused external morphological alterations to *C. albicans* and the degree of damage increased proportionally with the increase in time of treatment with NCs. Based on the SEM observations, it was evident that NCs have the ability to cause significant damages in the morphology of *C. albicans*.

**Figure 4.**
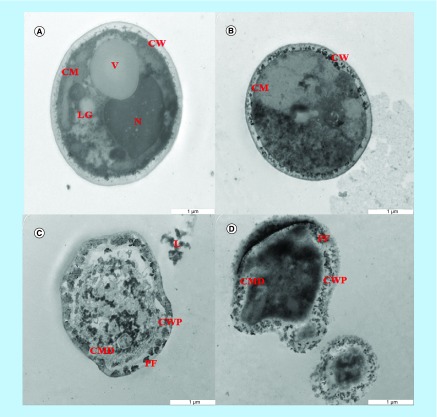
**Transmission electron microscopy micrograph of a cross section of the untreated and nanocapsule-treated cells of *Candida albicans*.** **(A)** Control cells of *C. albicans*; **(B)** 12 h *C. albicans* cells treated with 2 mg/ml of NCs; **(C)** 24 h *C. albicans* cells treated with 2 mg/ml of NCs and; **(D)** 36 h *C. albicans* cells treated with 2 mg/ml of NCs. ×6300 magnification used for all images. CM: Cell membrane; CMD: Cell membrane disruption; CW: Cell wall; CWP: Cell wall perturbation; L: Leakage; LG: Lipid granule; N: Nucleus; NC: Nanocapsule; PF: Pore formation; V: Vacuole.

From the SEM study, it can be observed that NCs caused severe surface ultrastructural damages. Further intramorphological damages providing evidence of this SEM observation were obtained by TEM evaluation on similarly treated *C. albicans*. Figure 4A shows the vehicle treated *C. albicans* (control) which are treated with deionized water only. The control cells showed normal intramorphological structure with various organelles of yeast cells such as nucleus, vacuole and lipid granule surrounded by the cytoplasm. The cytoplasm is surrounded by the cell membrane and cell wall which has a uniformed thickness. However, Figure 4B shows that there were changes in the intramorphological features of the yeast cells and lysis of the yeast cells that start to occur after 12 h of treatment with NCs. The internal organelles such as nucleus and vacuole were no longer seen. NCs have altered the cytoplasm of *C. albicans* cells as compared with the untreated control cells. The internal organelles were destroyed and dispersed which could have been due to the penetration of NCs into the yeast cells.

After 24 h of treatment of *C. albicans* with NCs (Figure 4C), severe alteration of the internal organelles was observed. Impartiality of cell membrane from the cell wall and formation of pores were noticed which caused the leakage of the intracellular contents. The leakage of intracellular contents resulted in some cytoplasmic contents found extracellularly. Besides that, the disorganization of the cell wall and disruption of cell membrane were also observed. Moreover, the yeast cells which had been treated with NCs for 36 h showed cell wall perturbation, cell membrane disruption and pore formation with a complete collapse of the cells (Figure 4D). The shrinkage of the cytoplasm from the cell wall resulted in the halo of the cell. The leakage of the intracellular granulated contents from the yeast cells due to pore formation on the cell wall was also discovered which eventually killed the cells. This shows that the NCs have the ability to cause alterations in intramorphological features of the *C. albicans* cells. The degree of changes deteriorated with the increase in time of treatment with NCs.

### *In vivo* antimicrobial activity of NCs

**Figure 5.**
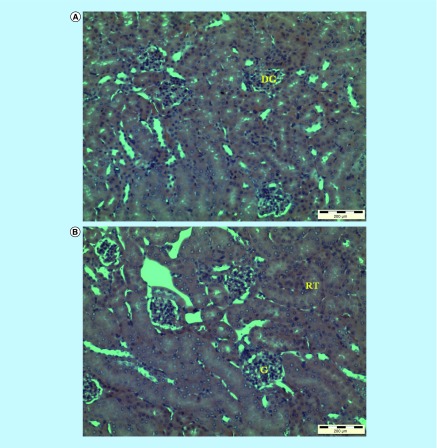
**Histomorphologies of the untreated and nanocapsule-treated kidney of mice infected with the *Candida albicans* (H & E staining; Magnification: x100).** DG: Damaged glomerulus; G: Glomerulus; H & E: Hemotoxylin and eosin; RT: Renal tubule.

Table 2 shows the mean of CFU/g of organs and CFU/ml of blood from Group 1 (control) and Group 2 (curative). A significant reduction (p < 0.05) in yeast cells was found in the kidney and blood samples evaluated in Group 2 animals that received a 2.5 g/kg body weight dose of the NCs. The presence of *C. albicans* cells in *in vivo* experiments was confirmed by using lactophenol cotton-blue staining method. A fivefold difference in CFU was observed between the kidneys and blood samples of mice from the treated group compared with those of the control group. The microscopic structures of the kidney depicted in Figure 5 show noticeable differences between Group 1 and Group 2. The microscopic examination reveals that the kidney from the Group 1 (Figure 5A) shows changes in cell structure or unfavorable effects with a damaged glomerulus when observed under the light microscope compared with NCs treated Group 2 (Figure 5B). The structure or coordination of cells in NCs treated kidney from mice in Group 2 is better when compared with that of the mice in Group 1. The results show that NCs are effective in inhibiting the *Candida* infection. Group 1’s mice which were infected with the *C. albicans* demonstrate damage to the kidney glomerulus and changes in the cell structure. However, Group 2’s mice that were treated with NCs do not reveal such damages to the kidney glomerulus and the cell structure is in a better shape probably because NCs cause the inhibition of *C. albicans* as observed in the reduction of CFU in the kidney and blood of Group 2.

**Table 2.** **Effects of nanocapsules on *Candida albicans* recovered from the kidney and blood of mice.**

**Group**	**Kidney (CFU/g)**	**Blood (CFU/ml of blood)**
Group 1 (control) intravenous *C. albicans* and intraperitoneal PBS	2.13 × 10^5^ ± 13,412	2.69 × 10^5^ ± 12,536

Group 2 (curative) intravenous *C. albicans* and intraperitoneal NCs	4.12 × 10^4^ ± 64*	4.99 × 10^4^ ± 53*

All values are colony-forming units (CFU/g organ or CFU/ml of blood) expressed as a mean ± standard error of the mean of three determinations.

*p < 0.05 compared with the control (Student *t*-test).

CFU: Colony-forming unit; NC: Nanocapsule; PBS: Phosphate-buffered saline.

### Inhibition of germ tube formation by NCs

**Figure 6.**
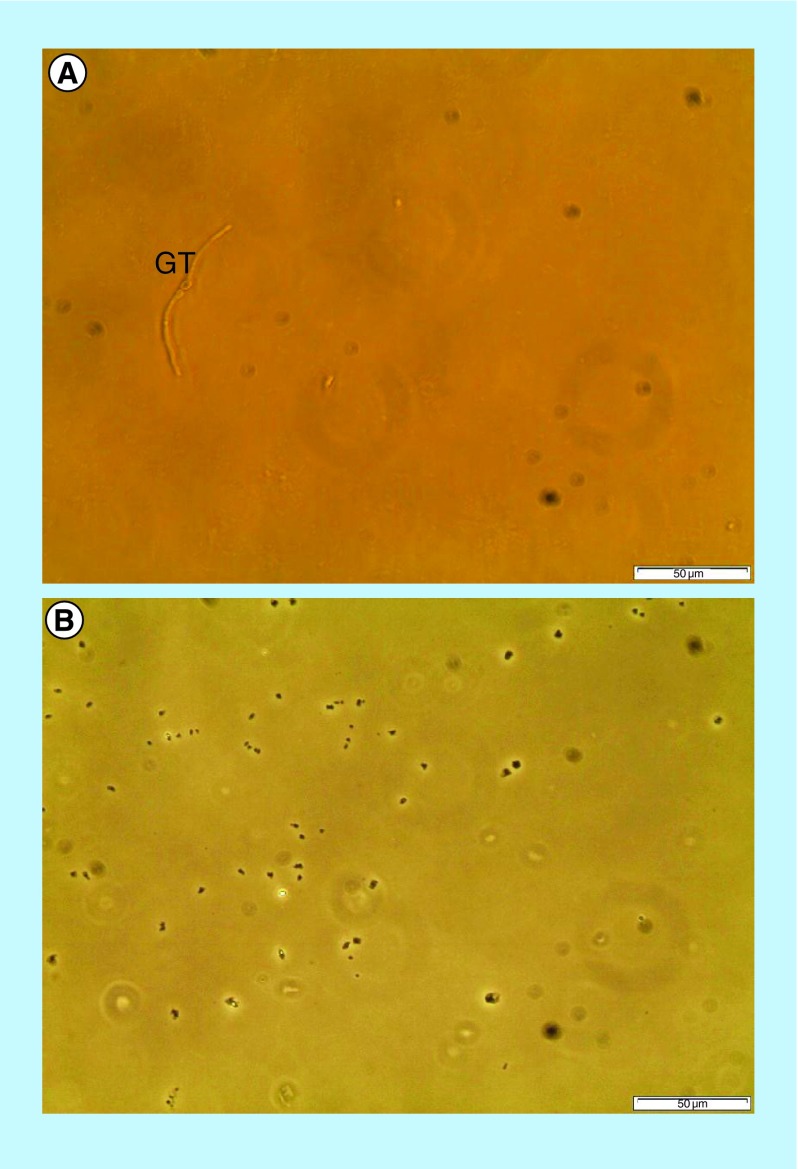
**Effect of the nanocapsule treatment on germ tube formation by *Candida albicans* (Magnification: x400).** **(A)** Untreated control *C. albicans* and; **(B)** Nanocapsule-treated *C. albicans*. GT: Germ tube.

The inhibition of germ tube formation study’s result is depicted in Figure 6. Figure 6A shows the germ tube formation by *C. albicans* after the incubation in fetal bovine serum for 1 h without the treatment of NCs (control). After 1 h of incubation, germ tube formation was observed using a phase-contrast microscopy. However, there is no germ tube formation seen after the treatment of the yeast cells with 2 mg/ml of NCs (Figure 6B). This finding showed that NC treatment had effectively inhibited the germ tube formation in *C. albicans*.

### Enhancement of the fungicidal properties of oral epithelial cells by NCs

**Figure 7.**
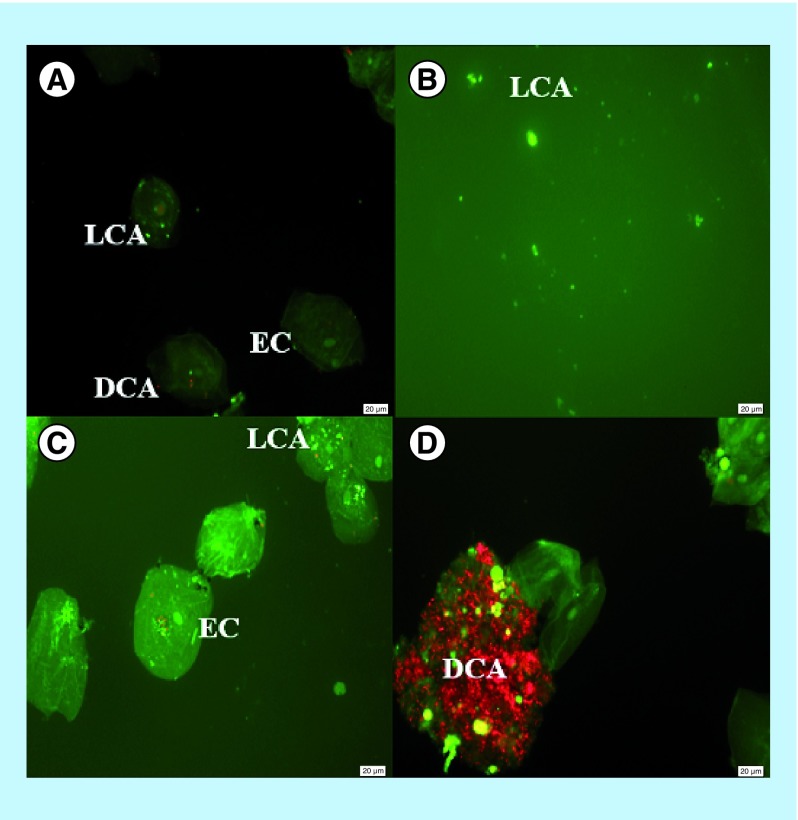
**Fluorescent images of vital staining of *Candida albicans* in the coculture of oral epithelial cells.** Whole unstimulated saliva was collected from healthy human volunteer, and epithelial cell-enriched populations were isolated and placed into the culture with *C albicans*. **(A)** Live (green) and dead (red) *C. albicans* with oral epithelial cells; **(B)** live *C. albicans* without oral epithelial cells; **(C)** fluorescent image of the phase-contrast image of untreated epithelial cells cultured with *C. albicans* (predominantly live cells) and; **(D)** fluorescent image of the phase-contrast image of epithelial cells cultured with *C. albicans* (predominantly dead cells) treated with 2 mg/ml nanocapsules. x400 magnification used for all images. DCA: Dead *Candida albicans*; EC: Epithelial cell; LCA: Live *Candida albicans*.

The epithelial cell–*C. albicans* coculture growth inhibition assay was performed in the presence or absence of NCs to confirm the enhanced inhibition of *C. albicans* by the cells and NCs. The vital staining of *C. albicans* in the coculture is shown in Figure 7. Figure 7A demonstrates the verification of the right staining of live and dead *C. albicans* cells in the presence of oral epithelial cells. Figure 7B shows the live (green) *C. albicans* cells following by the 1-h incubation without human oral epithelial cells and stained with fluorescein diacetate (which stains living cells green) and propidium iodide (which stains dead cells red). Figure 7C shows a fluorescent image of human oral epithelial cells and *C. albicans* coculture following the 1-h incubation that reveals the presence of more live (green) and less dead (red) *C. albicans* cells. This finding showed that the *C. albicans* cells were killed by epithelial cells only to a certain extent. Figure 7D shows the fluorescence image of NC-treated coculture of human oral epithelial cell and *C. albicans* following the 1-h incubation that reveals the predominantly dead *C. albicans* cells. *The presence* of *C. albicans* cells on oral epithelial cells was confirmed by using lactophenol cotton-blue staining method. This result confirmed that the NC treatment further enhances the killing of *C. albicans* by oral epithelial cell which caused increased death in the *C. albicans* cells compared with epithelial cell–*C. albicans* coculture without the treatment of NCs.

## Discussion

### Antimicrobial activity of NCs

The inhibitory effect of NCs on pathogenic yeasts *C. albicans* has been investigated in this study by using the disk diffusion method. Presence of a clear zone indicated the susceptibility of the yeast against NCs. The observed cleared antimicrobial zone might be produced by the spreading of the nanoparticles surrounding the disk in this qualitative assay. Interestingly, negative control disk without NCs has exhibited no inhibition zone. Since NCs showed antimicrobial properties against *C. albicans* USM-K1 by the disk diffusion method, the minimal inhibitory concentration was determined to identify the minimum concentration of NCs that could inhibit the yeast growth. The lowest concentration of peptide that completely inhibited the growth of microorganisms by visual inspection or growth percentage, that was less than 5% of negative control detection spectrophotometrically, was defined as MIC [34]. The lowest concentration of antibiotic, causing absence of growth after 16–20 h of incubation, is the MIC [19]. Both the effective chemotherapeutic agent and a suitable concentration to control an infection can be evaluated. The lowest concentration should be maintained at the sites of infection considering that it is the minimum concentration that can cure a particular disease [35]. The results of MIC can be correlated with known drug concentrations targeted at different body parts. This method is important to determine the dose of drugs for the treatment of diseases. MIC is the test commonly evaluated for most infections [36]. Typically, the bovine lactoferrin at 100 μg/ml or more caused inhibition of *C. albicans* [37]. The MIC of NCs in this study which is 500 μg/ml is higher due to the coating of bLf by alginate. The MIC of NCs is higher as compared with peptides in other research because NCs were produced from bLf which is a globular protein. For instance, a novel antimicrobial peptide derived from the modified N-terminal domain of bLf (L10) demonstrated MIC of 100 μg/ml against *C. albicans* [38]. In another study, a N-terminal peptide of bovine lactoferrin known as lactoferricin-B (LF-B) demonstrated MIC of only 10 to 40 μg/ml against *C. albicans* [39].

### Fungicidal effect of NCs at various concentrations over time

Following the determination of minimal inhibitory concentration of NCs, time–kill study was conducted at various concentrations to observe the prolonged anticandidal properties of NCs over a duration of time. The effect of NCs toward the growth profile of *C. albicans* was determined by OD or absorbance. This method is based on the principle that microbial cells scatter light which striked them. Considering that *C. albicans* cells in a population are approximately constant, the amount of light scattering is directly proportional to the biomass of cells which are indirectly related to the number of cells. The degree of light scattering of *C. albicans* in this study was measured by a spectrophotometer. Therefore, the population growth of *C. albicans* can be measured spectrophotometrically as long as the population is high enough to enable detectable turbidity [19].

Time–kill studies are usually employed in the search of new antimicrobial agents. They are popular in preclinical studies because they are relatively easy and cheap to be performed. Microbial regrowth after an initial reduction in the initial inoculum is a significant problem. Time–kill study provides descriptive (qualitative) information on the pharmacodynamics of antimicrobial agents. This technique provides valuable information about the cidal action in relation to the concentration of the test substance over time [40].

In this study, time–killing profile was conducted to further confirm the results of *in vitro* antimicrobial activities. The time–killing study revealed prolonged anticandidal activity when *C. albicans* was exposed to NCs at 0.5 MIC, MIC and 2 MIC, 4 MIC for 48 h. *In vitro* antimicrobial methods are controversial because the findings do not always correlate with the *in vivo* results or clinical outcome. Hence, this indicates that conventional MIC determinations are insufficient for detecting the effectiveness of antimicrobial agents against pathogens. Time–kill curves of *C. albicans* exposed to several NCs concentrations determine antimicrobial properties over a duration of time and can also compare the differences in antimicrobial properties of NCs with different MIC concentrations.

The results of this study indicated that NCs possess good anticandidal properties whereby concentration dependent killing was observed against the tested yeast strain. Interestingly, the potent anticandidal properties of NCs in time–killing studies were demonstrated by NCs with MIC, 2 MIC and 4 MIC showing a decrease in the viability OD_600_ = 2.2 in the evaluated strain. In any type of microbial infection, the first step in the investigation of an appropriate therapeutic agent is the assessment of the causative microorganism’s predisposition *in vitro* to potentially effective agents. Drugs that prove less effective *in vitro* are not likely to be considered for treatment. The outcomes of this study also demonstrated that NCs is active against clinical isolates judging from its MIC (500 μg/ml). Besides that, NCs at 2 MIC produced a viability decrease of OD_600_ = 2.2 against *C. albicans*. It is possible that the NCs concentration of 2 MIC could be achieved at the site of *C. albicans* infection considering that 1 g or more doses of NCs can be administered orally. Therefore, these data suggest the requirement for evaluating the *in vitro* and *in vivo* effectiveness of NCs against *C. albicans.* The findings of this study also clearly indicated the potential of NCs to be developed as a therapeutic agent against *C. albicans* infection. Therefore, it can be concluded that NCs have the ability to alter the growth profile of *C. albicans* at 0.5 MIC, MIC and 2 MIC compared with the control group. The growth profile also proved that the MIC of NCs obtained in this study was fungicidal as *C. albicans* was killed over the duration of 48 h. This phenomenon is important in drug development to ensure that pathogens are totally inhibited and eliminated.

### SEM & TEM observations

Subsequently, SEM and TEM were used to further study the *in situ* ultrastructural changes caused by NCs on *C. albicans* cells at various exposure time and its preliminary anticandidal mode of action. The SEM method is considerably beneficial compared with several other microscopic methods because it is 3D and almost the entire cell is sharply focused. Other advantages include higher magnification, larger depth of focus, greater resolution and easier sample preparation compared with TEM. However, a limitation of this method is that the sample must be in solid form [41]. On the other hand, the disadvantage of TEM is that only one cell is shown as the single cell which is considered as a representative for the particular treated group. The microscopic examinations of *C. albicans* using SEM displayed that cells treated with NCs seemed irregular in morphology with cell wall modifications and clear depression on the surface of cells. The TEM observations revealed irregular cell wall, ruptured cell membrane and dense cytoplasm without distinguished features. Such modifications could be triggered by the interference of NCs. The cell membranes of yeast form a barrier to the passage of small ions such as H^+^, K^+^, Na^+^ and Ca^2+^ and allow cells and organelles to control the entry and exit of different substances. The permeability of cell membranes is vital to many cellular functions such as maintenance of energy status of the cell, membrane coupled energy-transducing process, solute transport, regulation of cell metabolism and control of turgor pressure [42,43]. Cell leakage from both untreated and treated cells was almost similar in the first 12 h of incubation. This implies that NCs had minimal effect on the cell membranes during the first 12 h. Within 24–36 h, a significant increase in leakage from cells exposed to NCs could occur. In order for the cell leakage to occur, some alterations in the cell wall and cell membrane must occur. Interestingly, the exposure of *C. albicans* cells to NCs increased the permeability of cell membrane because of the disruption of cell wall and cell membrane structures. That facilitated the cell leakage as observed in the SEM and TEM studies. Therefore, the mechanism of NCs on *C. albicans* may be attributed to the disruption of the cell wall and cell membrane which was seen by both SEM and TEM which showed severe changes to the cell wall causing blebs on the surface and cell collapse. The mechanism of action of lactoferrin on yeast such as *C. albicans*, *Candida tropicalis* and *Candida krusei* is cell wall disruption [39] and severe alterations to the cell wall causing blebs on the surface, swelling and collapse of cells [44] are similar to the mechanism of NCs in this study. Besides that, binding of lactoferrin to cell walls resulting in membrane disruption and leakage of intracellular content [45] were also similar to the effect of NCs in this study. The observed mechanism of NCs on the cell membrane in this study was comparable to the damaging effects of lactoferrin on the cell membranes of *C. albicans*, *Escherichia coli* and *Staphylococcus aureus* as reported previously by using scanning electron microscopy [46].

The mechanism of action of antimicrobial peptides is different compared with conventional antifungal drugs. It has been discovered in recent years that mitochondria plays an important role leading to cell death [47]. A recent study by Andrés *et al.* [47] proposed the plasma membrane Pma1p (P3A-type ATPase) as the lactoferrin molecular target by using various yeast species. In accord with this findings, the treatment of hLf gradually increased the cytoplasmic ATP levels in *C. albicans*. They also demonstrated that the specific inhibitor of mitochondrial F-type ATPase proton pump (mtATPase) oligomycin is capable to nullify the antifungal activity of lactoferrin, signifying the important task of mtATPase in the apoptotic yeast cells death. They recommended that the lactoferrin aimed the plasma membrane Pma1p H ± ATPase, disturbing the cytoplasmic ion homeostasis (accumulation of H+ followed by effluxion of K+) and prompting a deadly mitochondrial malfunction. This early happening implicated a usual mitochondrial ATP synthase activity facilitate the ATP increase and following hypothetical mitochondrial proton flooding process. In brief, the authors concluded that the lactoferrin blocked the Pma1p H ± ATPase and thus encouraged an apoptotic-like cell death process in yeast. Participation of mitochondrial H ± ATPase (nonreturned) was important for the development of this apoptotic cell death in which the ionic homeostasis agitation appears to lead traditional nonionic apoptotic events. Hence, in this study, the mechanism of NCs on cell membrane causes pore formation and leakage of the intracellular components based on SEM and TEM studies and caused progression to cell death, possibly with the involvement of mitochondria.

### Inhibition of germ tube formation by NCs

The inhibition of germ tube formation was also carried out since the transformation of yeast to hyphae causes colonization and invasion of host tissues. *C. albicans* is a dimorphic fungi that can change from the yeast form in human to the mycelium form in the external environment in response to various environmental conditions such as nutrients, carbon dioxide tension, oxidation–reduction potentials and temperature [19]. The transformation of *C. albicans* cell morphology from yeast to hyphae is one of the most important characteristics that allows colonization, invasion and survival of *C. albicans* in the host tissues during an infection. The mode of growth of *C. albicans* is determined by environmental conditions. *C. albicans* can go through obvious changes in morphology due to environmental conditions that involve various complex pathways such as MAPK and cAMP–PKA pathways. Therefore, any interruption with the gene expression involved in MAPK pathway and cAMP–PKA pathway can obstruct filamentation in *C. albicans* [48]. Attachment of microorganisms to surfaces and hypha formation is dependent on various factors such as cell hydrophobicity, electrostatic forces and specific adhesins. The ALS (agglutinin-like sequence) gene family which encodes cell surface glycoproteins is the most important among the *C. albicans* adhesion molecules and hypha-specific genes. *ALS1*, *ALS3* and *HWP1* genes regulated by EFG1 control *C. albicans* morphological changes [48]. Serum is an external factor that has an effect on hypha formation of *C. albicans* [49,50]. Therefore, inhibition of *C. albicans* germ tube in this study by NCs could be due to the interference of MAPK or cAMP–PKA pathway.

In oral candidiasis, the pathogenic importance of yeast as compared with the filamentous form is not clearly understood. However, conditions that were found to be conducive for germ-tube formation increased attachment of yeast cells to oral epithelial cells [51,52]. The presence of germ tubes increases the virulence of *C. albicans* in vaginal candidiasis [52,53]. Hence, the inhibition of germ tube formation by NCs in this study decreases adherence and virulence of *C. albicans* cells to oral epithelial cells and reduces the occurence of oral candidiasis.

### *In vivo* anticandidal activity of NCs

*In vivo* studies are important to prove the efficacy of *in vitro* findings obtained earlier in this study. Moreover, *in vivo* animal model system has similar basic biology and physiology of both normal functions and malfunctions as that in mice which yield important clues that could translate to human biology. In *in vivo* studies, the drug consumed is subjected to the physiological conditions in the body of the organism being studied which could be a limitation in the drug development process. NCs administered in mice in this study could be suppressed by physiological conditions as NCs enter directly into the blood stream, then onto the infected site of the mice. Hence, the current study was conducted to observe the effectiveness of NCs in the treatment of mice induced systemically with candidiasis. Considering the fact that systemic candidiasis infection is caused by *C. albicans*, this study was conducted on the systemic organs of mice. The advantage of studying animals instead of human beings is the ability to monitor animals and the environment [54]. Animal models are effective to be employed for the study of pathogenesis, host response and treatment of oral candidiasis infection [55]. Besides that, the utilization of animal models also excludes the need to obtain tissue samples from the infected area in humans.

Although various species of animals such as rats, rabbits and monkeys have been utilized, mice have been chosen in this study because mice are cheap, easily available and simple to handle [54]. An antimicrobial agent given orally has to fulfill several criteria such as the ability to enter the bloodstream, distribution in the subcellular areas, expression of antimicrobial properties at the site of infection and others in order to demonstrate optimum antimicrobial properties. It has to be taken into consideration that mice do not usually get infected by *C. albicans* compared with humans, and therefore, systemic infection needs to be induced experimentally [54]. In this study, the systemic infection has been induced successfully and has been shown by a histopathological examination by H&E staining. Blood and kidneys were chosen for the enumeration of the number of yeast cells because *Candida* infection induced systemically is carried by the blood stream to the kidneys where filtration takes place. The findings of this *in vivo* study showed that the NCs have the ability to reduce pathogenic *C. albicans* cells from both blood and kidney of infected mice. Therefore, the *in vivo* study proves the efficacy of *in vitro* findings obtained in this study. Besides that, the CFU results were parallel to the kidney histopathological examination. The findings of this study clearly show that the NCs were very effective in the treatment of mice infected systemically by *C. albicans* based on the CFU results and histopathological examination.

### Enhancement of anticandidal properties of oral epithelial cells by NCs

Apart from the *in vivo* studies, vital staining method was conducted to observe the further enhancement of the anticandidal properties of oral epithelial cells by NCs which will facilitate the strong anticandidal activity of oral and vaginal epithelial cells to inhibit the growth of *C. albicans*. Epithelial cells from the oral mucosa act as a physical barrier to prevent the entry of pathogens. Besides that, epithelial cells secrete various cytokines as a response to microorganisms, express major histocompatibility complex class II antigens, process and presentation of foreign antigens, and generate antibacterial and antifungal substances such as defensins, histatins and calprotectin. In other words, epithelial cells play multiple roles in the mucosal immunity [56,57].

Recent studies have reported the ability of oral and vaginal epithelial cells to inhibit the growth of *C. albicans in vitro* probably as an innate immune response. Oral epithelial cells have stronger anticandidal activity compared with vaginal epithelial cells [33]. Similarly, oral epithelial cells in this study also caused inhibition of *C. albicans*. It has been reported that cell contact by live epithelial cells with *C. albicans* is essential for vaginal and oral epithelial cell anticandidal activity without depending on soluble factors [33]. Research on the specific portions of oral epithelial cells showed that the carbohydrate moiety in epithelial cells has a role in the anticandidal properties but not the surface proteins or lipids. This phenomenon was proven by the removal of anticandidal activity after the treatment of epithelial cells with periodic acid. Studies have reported that proteins, glycoproteins or phospholipids were not involved the anticandidal activity except for carbohydrate. Besides that, the anticandidal activity of vaginal epithelial cells was also dependent on the carbohydrate moiety [33].

This study showed that antimicrobial peptides produced by epithelial cells were able to kill *C. albicans* only to a certain extent since not many dead cells (red cells) were observed when the epithelial cells were incubated with the yeast cells. However, more dead cells (red cells) were seen when NCs were added into the epithelial cells-yeast coculture indicating that NCs further enhanced the anticandidal effect of epithelial cells. Hence, it was suggested that NCs can contribute an important role against yeast infection as it has the ability to kill *C. albicans*. As such, NCs can be incorporated in milk and other dairy products such as ice cream or yogurt as a functional food to eliminate yeast infection through oral administration. Moreover, it was reported that the bovine lactoferrin derived antimicrobial peptides were suitable to be added to milk and other dairy products [58]. Interestingly, the inhibition of germ tube formation properties of NCs also may decrease the virulence of *C. albicans* cells to oral epithelial cells.

## Conclusion

In summary, the research presented in this article conclusively demonstrates the *in vitro* and *in vivo* anticandidal potential of NCs. NCs are proven to be fungistatic, fungicidal and anticandidal agent for *C. albicans*. Furthermore, NCs may have potential to treat candidiasis although this use will require additional detailed investigation.

## Future perspective

Lactoferrin is a multifunctional iron-binding glycoprotein present in mammalian milk. Despite various research reporting the anticandidal effects of lactoferrin, encapsulated lactoferrin has never been tested for anticandidal activity in detail. Therefore, there is a need to evaluate encapsulated lactoferrin to understand in detail its anticandidal activity. In view of this, alginate-enclosed chitosan–calcium phosphate-loaded Fe-bLf NCs were prepared and tested for *in vitro* and *in vivo* anticandidal activity against *C. albicans*. Collectively, all evidence from this study showed that NCs have a potential for the development as a novel therapeutic agent for the treatment of *C. albicans* infection. The next steps for this research are to determine the appropriate curative concentration of NCs for human application and to perform the clinical trials to treat yeast infection in human. Lastly, NCs also need to go through detailed toxicity profiling for the better clarification of their appropriateness for the treatment of *C. albicans* infection in human.

**Summary points**

**Background**

- The discovery of antifungal agents remains an important scientific challenge.
- Lactoferrin is a multifunctional iron-binding glycoprotein. It has been shown to inhibit a wide range of fungals due to cell wall perturbation.
- Encapsulated lactoferrin has never been tested for anticandidal activity in detail.

**Aim**

- This research was conducted to investigate the *in vitro* and *in vivo* anticandidal activity of Alginate/EUDRAGIT^®^ S 100-enclosed chitosan–calcium phosphate-loaded Fe-bovine lactoferrin nanocapsules (NCs).

**Results**

- NCs showed a good antimicrobial activity against *C. albicans* with MIC value of 500 μg/ml.
- NCs disrupted the cell wall and cell membrane of *C. albicans* cells.
- The growth profile study confirmed the fungicidal effect of the NCs on *C. albicans* by changing the normal growth profile.
- *In vivo* anticandidiasis study proved the efficacy of NCs in animal models.
- NCs enhanced the natural killing of C. *albicans* properties by epithelial cells.

**Conclusion**

- This is the first detailed work done on *in vitro and in vivo* anticandidal activities of NCs. NCs have effective anticandidal properties and have the potential as a therapeutic agent against candidiasis.
